# The complete chloroplast genome and phylogenetic analysis of *Potentilla sischanensis* Bunge ex Lehm

**DOI:** 10.1080/23802359.2021.1991244

**Published:** 2021-10-20

**Authors:** Yupeng Guo, Buqing Yao, Wenting Da, Feichao Du, Mengran Yuan, Junqiao Li

**Affiliations:** a Qinghai Provincial Key Laboratory of High Value Utilization of Characteristic Economic Plants, College of Ecological Environment and Resources, Qinghai Nationalities University, Xining, Qinghai, P. R. China; b Key Laboratory of Cold Regions Restoration Ecology, Qinghai Province, and Key Laboratory of Adaptation and Evolution of Plateau Biota, Northwest Institute of Plateau Biology, Chinese Academy of Sciences, Xining, People’s Republic of China

**Keywords:** *Potentilla sischanensis* Bunge ex Lehm., chloroplast genome, phylogenetic analysis

## Abstract

*Potentilla sischanensis* Bunge ex Lehm. is a widespread perennial herb in north China. The plant has little yellow flowers, and the petioles are white-tomentose and sparsely villous. To determine the chloroplast genome, total genomic DNA was extracted from fresh leaves and sequenced. The complete chloroplast genome was assembled and annotated. The chloroplast genome of this plant is a circular form with a length of 156,240 bp, including a large single-copy region (LSC, 85,748 bp), a small single-copy region (SSC, 18,566 bp), and two inverted repeats (IRs, 25,963 bp). A total of 132 genes were predicted, comprising 87 encoded proteins, 8 rRNAs and 37 tRNAs. The evolutionary history indicates that *P. sischanensis* was grouped within *Potentilla* and formed a clade with *Potentilla chinensis* and *Potentilla stolonifera* with a 100% bootstrap support value. The complete cp genome can serve as a reference for future studies on molecular biology, evolution, population genetics, taxonomy and resource protection.

*Potentilla sischanensis* Bunge ex Lehm., belonging to Rosaceae, is a perennial herb that is 10–330 cm tall with little yellow flowers and petioles that are white-tomentose and sparsely villous. This plant is widespread in northern China (Delectis Florae Reipublicae Popularis Sinicae Agendae Academiae Sinicae Edita 1989). For containing bioactive constituents, most of *Potentilla* species have therefore been used extensively in traditional medicine. Extracts from these plants have been used to cure toothache, inflammation of the throat, and ulcers of the mouth in Europe and to treat diarrhea, hepatitis, rheuma, scabies and detoxification in China (Tomczyka and Latté 2009). Extracts from some species can also delay the process of carcinogenesis (Ganguly et al. 2019; Kowalik et al. 2020). The extract from *Potentilla rugulosa* leaves has been identified as a potential functional food supplement for preventing the development of obesity (Choi et al. 2020). Among the many studies that have been performed on *Potentilla* plants, very few studies have been performed on *P. sischanensis*, except for one phylogenetic study (Feng et al. 2017). Here, we report the complete chloroplast (cp) genome of *P. sischanensis* and analyze its phylogenetic relationship with other related species.

Samples were collected from the Qilian Mountains (36°34′21″N, 101°48′44″E) in Qinghai Province. A specimen was deposited at the College of Ecological Environment and Resources, Qinghai Nationalities University (https://shxy.qhmu.edu.cn/, Junqiao Li, email: ljqlily2002@126.com) under voucher number HCEERQNU-20200517001. Total genomic DNA was extracted from the fresh leaves of a sample with a Rapid Plant Genomic DNA Isolation Kit. Paired-end libraries with an average length of 500 bp were constructed and sequenced on the Illumina HiSeq 4000 platform (Sangon Biotech (Shanghai) Co., Ltd.). The complete cp genome was assembled via NOVOPlasty 3.7.2 (Dierckxsens et al. 2017) with *Potentilla freyniana* Bornm. (GenBank accession no. MK472813.1) as the reference genome. The complete assembled genome was annotated via PGA (Qu et al. 2019).

The complete cp genome of *P. sischanensis* (GenBank accession no. MW678838.1) has a typical quadripartite form with a length of 156,240 bp and is composed of a large single-copy region (LSC, 85,748 bp), a small single-copy region (SSC, 18,566 bp), and two inverted repeats (IRs, 25,963 bp). The genome has a GC content of 37%. A total of 132 genes were predicted in this cp genome, comprising 87 encoded proteins, 8 rRNAs and 37 tRNAs.

Phylogenetic analysis was performed based on the complete cp genomes of *P. sischanensis* and 33 other related species in Rosaceae with two species in Rhizophoraceae as outgroup. The genome-wide alignment was constructed by HomBlocks (Bi et al. 2018), the evolutionary history was inferred using the maximum likelihood (ML) method by IQ-TREE 1.6.12 under the GTR + F + R3 model (Nguyen et al. 2015; Kalyaanamoorthy et al. 2017), and the output file was edited in MEGA X (Kumar et al. 2018). Bootstrap (BS) values were calculated by UFBoot2 from 1000 replicates (Hoang et al. 2018). As expected, *P. sischanensis* was grouped within *Potentilla* and formed a clade with *Potentilla chinensis* Ser. and *Potentilla stolonifera* Lehm. ex Ledeb. with a 100% BS support value (Figure 1). The complete cp genome of *P. sischanensis* can serve as a reference for future studies on molecular biology, evolution, population genetics, taxonomy and resource protection.

**Figure 1.**
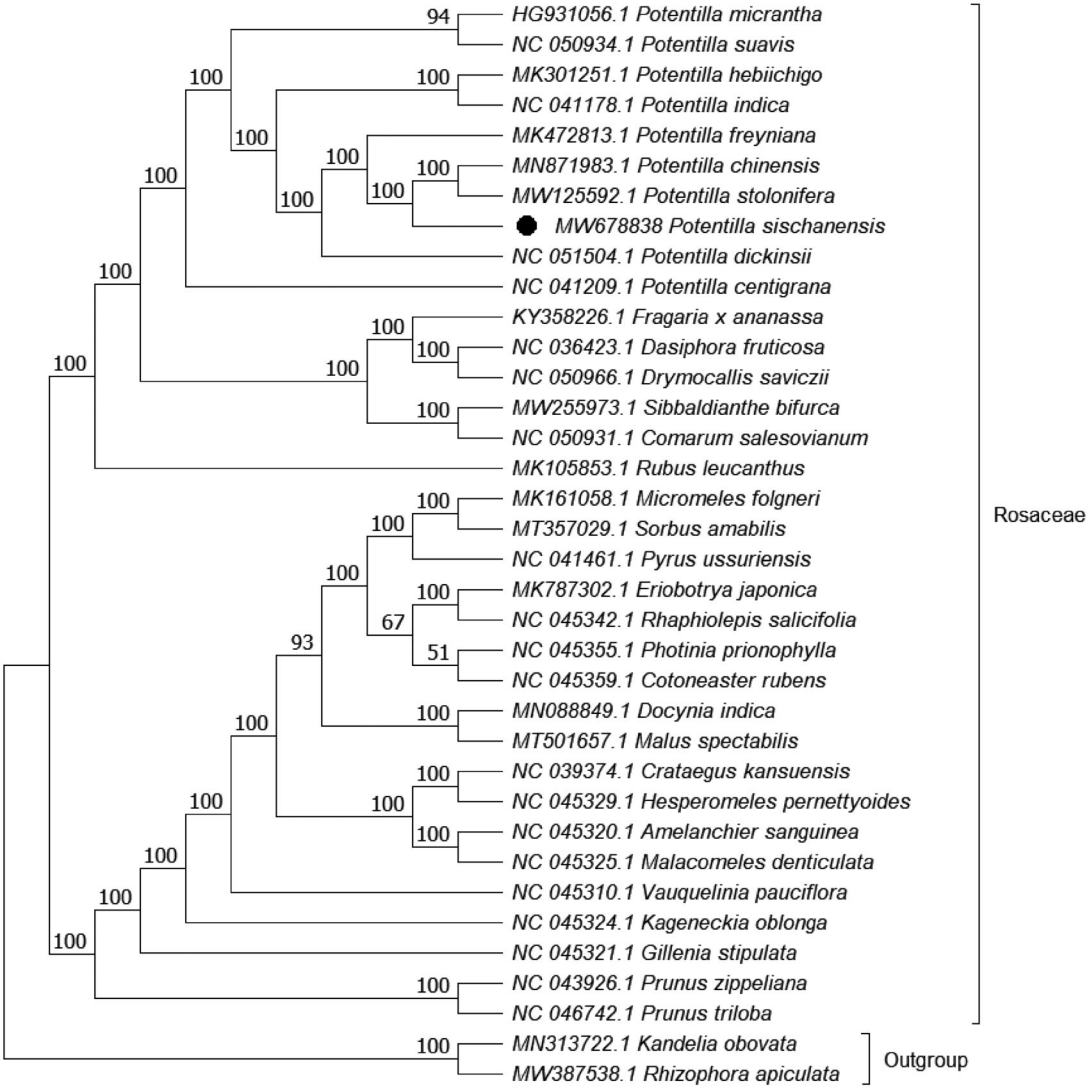
ML phylogenetic tree based on 36 species chloroplast genomes was constructed using IQ-TREE 1.6.12. Numbers on each node are bootstrap support values from 1000 replicates.

## Data Availability

The genome sequence data that support the findings of this study are openly available in GenBank of NCBI at (https://www.ncbi.nlm.nih.gov/nuccore/MW678838) under the accession no. MW678838. The associated BioProject, SRA, and Bio-Sample numbers are PRJNA725277, SRR14328193, and SAMN 18875820, respectively.

## References

[CIT0001] Bi GQ, Mao YX, Xing QK, Cao M. 2018. HomBlocks: a multiple-alignment construction pipeline for organelle phylogenomics based on locally collinear block searching. Genomics. 110(1):18–22. 28780378 10.1016/j.ygeno.2017.08.001

[CIT0002] Choi SI, Lee JS, Lee S, Sim WS, Kim YC, Lee OH. 2020. *Potentilla rugulosa* Nakai extract attenuates bisphenol A-, S- and F-induced ROS production and Di_erentiation of 3T3-L1 preadipocytes in the absence of dexamethasone. Antioxidants. 9 (2):113. 10.3390/antiox9020113 PMC7071078 32012803

[CIT0003] Delectis Florae Reipublicae Popularis Sinicae Agendae Academiae Sinicae Edita. 1989. Flora reipublicae popularis sinicae. Vol. 37. Beijing: Science Press; p. 286.

[CIT97242347] Dierckxsens N, Mardulyn P, Smits G. 2017. NOVOPlasty: de novo assembly of organelle genomes from whole genome data. Nucleic Acids Res. 45(4):e18 doi:10.1093/nar/gkw955. PMC: 28204566 28204566 PMC5389512

[CIT0004] Feng T, Moore MJ, Yan MH, Sun YX, Zhang HJ, Meng AP, Li XD, Jian SG, Li JQ, Wang HC. 2017. Phylogenetic study of the tribe Potentilleae (Rosaceae), with further insight into the disintegration of Sibbaldia. J Syst Evol. 9999(9999):1–15.

[CIT0005] Ganguly B, Chaudhary A, Dakhar H, Singh IP, Chatterjee A. 2019. Methanolic extract of *Potentilla fulgens* root and its ethyl-acetate fraction delays the process of carcinogenesis in mice. Sci Rep. 9(1):16985. 31740710 10.1038/s41598-019-53747-5 PMC6861273

[CIT0006] Hoang DT, Chernomor O, von Haeseler A, Minh BQ, Vinh LS. 2018. UFBoot2: improving the ultrafast bootstrap approximation. Mol Biol E. 35(2):518–522. 10.1093/molbev/msx281 PMC5850222 29077904

[CIT0007] Kalyaanamoorthy S, Minh BQ, Wong TKF, von Haeseler A, Jermiin LS. 2017. ModelFinder: fast model selection for accurate phylogenetic estimates. Nat Methods. 14(6):587–589. 28481363 10.1038/nmeth.4285 PMC5453245

[CIT0008] Kowalik K, Paduch R, Strawa JW, Wiater A, Wlizło K, Waśko A, Wertel I, Pawłowska A, Tomczykowa M, Tomczyk M. 2020. *Potentilla alba* extracts affect the viability and proliferation of non-cancerous and cancerous colon human epithelial cells. Molecules. 25(13):3080. 10.3390/molecules25133080 PMC7411782 32640760

[CIT0009] Kumar S, Stecher G, Li M, Knyaz C, Tamura K. 2018. MEGA X: molecular evolutionary genetics analysis across computing platforms. Mol Biol Evol. 35(6):1547–1549. 29722887 10.1093/molbev/msy096 PMC5967553

[CIT0010] Nguyen LT, Schmidt HA, von Haeseler A, Minh BQ. 2015. IQ-TREE: a fast and effective stochastic algorithm for estimating maximum-likelihood phylogenies. Mol Biol Evol. 32(1):268–274. 25371430 10.1093/molbev/msu300 PMC4271533

[CIT0011] Qu XJ, Moore MJ, Li DZ, Yi TS. 2019. PGA: a software package for rapid, accurate, and flexible batch annotation of plastomes. Plant Methods. 15:50. 31139240 10.1186/s13007-019-0435-7 PMC6528300

[CIT0012] Tomczyk M, Latté KP. 2009. Potentilla – a review of its phytochemical and pharmacological profile. J Ethnopharmacol. 122(2):184–204. 19162156 10.1016/j.jep.2008.12.022

